# Change in Growth Differentiation Factor 15, but Not C-Reactive Protein, Independently Predicts Major Cardiac Events in Patients with Non-ST Elevation Acute Coronary Syndrome

**DOI:** 10.1155/2014/929536

**Published:** 2014-04-15

**Authors:** Alberto Dominguez-Rodriguez, Pedro Abreu-Gonzalez, Idaira F. Hernandez-Baldomero, Pablo Avanzas, Francisco Bosa-Ojeda

**Affiliations:** ^1^Department of Cardiology, Hospital Universitario de Canarias, Ofra s/n La Cuesta, 38320 Tenerife, Spain; ^2^Instituto Universitario de Tecnología Biomédicas, Ofra s/n La Cuesta, 38320 Tenerife, Spain; ^3^Department of Physiology, University of La Laguna, Ofra s/n La Cuesta, 38320 Tenerife, Spain; ^4^Hospital Universitario Central de Asturias, Area del Corazón, 33006 Oviedo, Spain

## Abstract

Among the numerous emerging biomarkers, high-sensitivity C-reactive protein (hsCRP) and growth-differentiation factor-15 (GDF-15) have received widespread interest, with their potential role as predictors of cardiovascular risk. The concentrations of inflammatory biomarkers, however, are influenced, among others, by physiological variations, which are the natural, within-individual variation occurring over time. The aims of our study are: (a) to describe the changes in hsCRP and GDF-15 levels over a period of time and after an episode of non-ST-segment elevation acute coronary syndrome (NSTE-ACS) and (b) to examine whether the rate of change in hsCRP and GDF-15 after the acute event is associated with long-term major cardiovascular adverse events (MACE). Two hundred and Fifty five NSTE-ACS patients were included in the study. We measured hsCRP and GDF-15 concentrations, at admission and again 36 months after admission (end of the follow-up period). The present study shows that the change of hsCRP levels, measured after 36 months, does not predict MACE in NSTEACS-patients. However, the level of GDF-15 measured, after 36 months, was a stronger predictor of MACE, in comparison to the acute unstable phase.

## 1. Introduction

Increasingly, cardiac biomarkers have provided important information in predicting short-term and long-term risk profiles in patients with acute coronary syndromes (ACS), particularly when they are used in combination [1]. Among the numerous biomarkers, high-sensitivity C-reactive protein (hsCRP) has received widespread interest and a large database has been accumulated on their potential role as predictor of cardiovascular events [2, 3]. Growth-differentiation factor-15 (GDF-15) is one of more than 40 members of the transforming growth factor-*β* superfamily and it was originally identified in activated macrophages [4]. Accumulating evidence indicates that circulating levels of GDF-15 are associated with the risk of death and myocardial infarction, independent of clinical variables and other biomarkers, including hsCRP and cardiac troponins [5, 6].

The inflammatory response triggered in the ACS setting is the cumulative result of preexisting, low-grade inflammation in vulnerable atherosclerotic plaques and the ongoing myocardial ischemic damage during the progression of an acute coronary event [7]. Consistently, the magnitude of the inflammatory response as reflected by the peripheral levels of inflammatory markers is largely determined by the temporal interval between symptom onset and the time point of biochemical measurement. Optimal interpretation of the elevated circulating levels of biomarkers would, therefore, require knowledge of their release curves and consideration of the time point of blood sampling [8, 9]. However, the long-term temporal changes of hsCRP levels and the relation between the changes of GDF-15 levels have not been examined, after an episode of non-ST-segment elevation ACS (NSTE-ACS).

Consequently, the aims of our study were to

1. describe changes of hsCRP and GDF-15 levels over time after an episode of NSTE-ACS,
2. examine whether changes of hsCRP and GDF-15 levels are associated with long-term major cardiovascular adverse events (MACE).

## 2. Methods

### 2.1. Study Population

Three hundred and eighty consecutive ACS patients were admitted to the coronary care unit of a university hospital. One hundred and twenty-five patients were excluded from analysis for the following reasons: patients with a history of systemic inflammatory diseases, such as infections or autoimmune disorders, neoplastic or haematological disease, administration of anti-inflammatory or immune-suppressive drugs, and surgical procedures or trauma in the preceding 3 months, patients with an equivocal or uninterpretable electrocardiogram, including left bundle branch block or persistent ST-segment elevation due to a myocardial infarction and patients with significant changes in medical therapy during followup. Thus, 255 NSTE-ACS patients were included in the study.

Patients were followed up for three years regarding the occurrence of MACE (death, myocardial infarction, and unstable angina (Class IIIb)). Therapeutic management during hospitalisation and in the outpatient clinic was left to the discretion of the attending cardiologist, according to the patients' clinical course, standard institutional protocols, and current guidelines [10]. The Ethics Committee of our institution approved the research protocol, and all patients gave written, informed consent for inclusion in the study.

### 2.2. Biochemical Analysis

Serial venous blood samples were obtained on admission from 8 am to 3 pm, to avoid the diurnal variation of inflammatory biomarkers reported by our group [11]. Blood samples were also obtained on a follow-up evaluation 36 months after admission. Serum samples were obtained by centrifugation, after the formation of the clot of blood, and stored at −70°C for subsequent analyses.

Concentrations of the serum hsCRP were measured, by an ultrasensitive, enzyme-linked, immunosorbent assay kit (DRG Instruments GmbH, Germany). In this enzyme-linked immunosorbent assay, the lowest detection limit of hsCRP was 0.010 mg/L. Coefficients of variation were 5.12% and 11.6% for intra- and interassay variabilities, respectively. Serum GDF-15 concentrations were measured using a commercially, enzyme-linked immunosorbent assay (BioVendor GmB, Heidelberg, Germany). In this assay, the lowest detection limit of GFD-15 is 30.2 pg/mL. Coefficients of variation were 4.3% and 7.8% for intra- and interassay variability, respectively. Troponin I was determined immune enzymatically, using a technique based on sandwich ELISA (Boehringer Mannheim, Germany). Coefficients of variation were 2.2% and 5.9% for intra- and interassay variabilities, respectively.

All other biochemical measurements were performed in the biochemistry laboratory of our hospital from the samples obtained at baseline, using standard methods. Personnel, blinded to patient's baseline characteristics and clinical outcomes, carried out all measurements.

### 2.3. Statistical Analyses

Results for normally distributed continuous variables are expressed as mean ± SD; nonnormally distributed continuous variables are presented as median and interquartile range. Categorical data is expressed as a percentage. Analysis of normality of the continuous variables was performed with the Kolmogorov-Smirnov test. Unpaired 2-tailed *t*-tests and the Mann-Whitney *U*-test assessed differences between the groups for continuous variables, as appropriate. Categorical data and proportions were analysed by use of *χ* ^2^ or Fisher's exact test when required. GDF-15 and hsCRP levels had a nonnormal distribution and were, therefore, logarithmically transformed before regression analysis to fulfill the conditions required for this type of analysis.

The information regarding the appearance of the endpoint, combined at a 36-month followup, was available for all patients included in the study. In patients who died during the 36-month followup period, the blood sample was not available, so we evaluated independent predictors of unstable angina (class IIIb) and myocardial infarction (combined primary endpoint). We defined the value delta, as a value that represents the difference between the concentrations of inflammatory markers at admission and at 36-month followup.

Independent predictors of changes were identified by multiple linear regression analysis and multivariable regression analysis, as appropriate. Tested covariates included sex, age, current smoking, diabetes mellitus, hypertension, dyslipidemia, coronary revascularisation, left ventricular ejection fraction, and troponin I. Delta GDF-15 concentrations were introduced into the multivariate model as a binary variable, considering the median as the cut-off value. Backward stepwise selection was used in multivariate analysis to derive the final model for which significance levels of 0.1 and 0.05 were chosen to exclude and include terms, respectively. Differences were considered to be statistically significant if the null hypothesis could be rejected with >95% confidence. The SPSS 15.0 statistical software package (SPSS Inc., Chicago, IL, USA) was used for all calculations.

## 3. Results

Demographic and clinical data of patients with and without MACE are presented in Table 1. After 36 months of followup, the combined endpoint ((cardiac death (7 patients), myocardial infarction (3 patients), and unstable angina class IIIB (35 patients)) appeared in 45 patients (17.6%). There were no significant differences in age, sex, cardiovascular risk factors, TIMI risk score, severity of coronary artery disease, treatment, and standard biochemical results between the two groups.

Regarding inflammatory biomarkers, we found no differences between both groups in levels of hsCRP at admission and after a three-year followup (Table 2). However, delta hsCRP concentrations were higher in patients who developed MACE compared to patients who did not (*P* = 0.01) (Table 2). We found significant differences in the GDF15 levels after the three-year followup between both groups (*P* < 0.001) (Table 2). Moreover, delta GDF-15 concentrations were higher in patients who developed MACE (*P* < 0.001) (Table 2).

Multivariate analysis showed that delta GDF-15 (OR = 52.3, CI 95% 7-388.5, *P* < 0.001) was the unique independent predictor of the combined endpoint (class IIIB unstable angina and myocardial infarction) at 36-month followup. Variables such as age (*P* = 0.15), sex (*P* = 0.23), smoking (*P* = 0.33), diabetes mellitus (*P* = 0.24), hypertension (*P* = 0.55), dyslipidemia (*P* = 0.57), revascularization (*P* = 0.70), left ventricular ejection fraction (*P* = 0.50), and troponin I (*P* = 0.15) were not independent predictors of MACE.

## 4. Discussion

The results from our study demonstrate different patterns of release of the hsCRP and GDF-15 with, over time, between patients with NSTE-ACS. Moreover, this is the first study to show that the changes in GDF-15, over time, are of prognostic relevance in NSTE-ACS patients.

GDF-15 is emerging as a prognostic biomarker in patients with ACS. The predictive value of GDF-15 measured on admission has been investigated in the two large NSTE-ACS populations: the Global Utilisation of Strategies to Open Occluded Arteries IV (GUSTO-IV) and Fast Revascularisation during Instability in Coronary Artery Disease II (FRISC II) cohorts [5, 12]. In another study, Damman et al. have evaluated the long-term prognostic value of GDF-15, regarding death or myocardial infarction in NSTE-ACS patients. They have shown that the Kaplan-Meier curves diverged early and continued to diverge up to five years [13].

In a recent study, the circulating concentration of GDF-15 was measured at baseline (*n* = 1734) and at 12 months (*n* = 1517) in patients randomised in the Valsartan Heart Failure Trial (Val-HeFT) [14]. They demonstrated increases in GDF-15 over 12 months, which were independently associated with the risks of future mortality and first morbid event also, after adjustment for clinical prognostic variables, B-type natriuretic peptide, hsCRP, and high-sensitivity troponin T and their changes.

In another study recent, Eggers and colleagues analysed GDF-15 concentrations in participants from the Prospective Investigation of the Vasculature in Uppsala Seniors (PIVUS) study. Measurements were performed at 70 and 75 years of age. They demonstrated that the GDF-15 concentrations and their changes over time are powerful predictors of mortality in elderly community-dwelling individuals [15].

In the results of our study, we found that NSTE-ACS patients, who developed MACE, displayed higher levels of GDF-15 at the 36-month followup than at admission. Furthermore, we were able to show that delta GDF-15 is associated with adverse outcomes, independently of established clinical and biochemical risk markers. Our results support the notion that GDF-15 integrates information on several relevant aspects and pathways in cardiovascular disease. The prominent antiapoptotic, antihypertrophic, and anti-inflammatory actions of GDF-15 in cardiovascular disease models indicate that this cytokine exerts protective effects in the context of acute cardiovascular injury [16]. Whether chronic increases in GDF-15 concentrations in NSTE-ACS patients play an adaptive or maladaptive role remains to be investigated.

In relation with hsCRP in our study, we measured hsCRP in two points, at admission and at 36 months. The delta value or rate of change of the hsCRP was useful to differentiate the group of patients with worse clinical outcome during 36 months of followup. However, after adjusting by different confounders, we have not demonstrated that delta hsCRP can predict MACE in NSTE-ACS patients. Recently, in a study by Karakas et al., they serially measured hsCRP concentrations in up to 6 blood samples, taken at monthly intervals from 200 postmyocardial infarction patients, who participated in the AIRGENE study. The results demonstrate considerable stability and good reproducibility for serial hsCRP measurements [17].

The implementation of hsCRP measurement into clinical practice requires sound data on the reliability of such measurement [11]. Data is still scarce for the long-term analytical variation of hsCRP measurement in patients with cardiovascular disease.

## 5. Conclusion

The present study shows that the rate of change of hsCRP measured at the 36-month followup was not predicting long-term MACE in NSTE-ACS patients. However, the delta GDF-15 at the 36-month followup seems to be a stronger predictor of MACE than during an acute unstable phase.

## Figures and Tables

**Table 1:** Clinical variables of non-ST-segment elevation acute coronary syndrome patients with and without MACE at 36-month followup.

Variable	MACE		*P* value
	Yes (*n* = 45)	No (*n* = 210)	
Age (years)	68 ± 11	66 ± 11	0.24
Men	25 (55.6%)	138 (65.7%)	0.23
Hypertension (>140/90 mmHg)	32 (71.1%)	133 (63.3%)	0.39
Hypercholesterolemia (>5.17 mmol/L)	19 (42.2%)	99 (47.1%)	0.62
Smokers	31 (68.9%)	121 (57.6%)	0.18
Diabetes	14 (31.1%)	75 (35.7%)	0.61
TIMI risk score			0.47
2	9 (20%)	35 (16.7%)	
3	9 (20%)	74 (35.2%)	
4	16 (35.6%)	58 (27.6%)	
5	10 (22.2%)	38 (18.1%)	
6	1 (2.2%)	5 (2.4%)	
Coronary artery disease			0.35
1 vessel	22 (4.9%)	115 (54.8%)	
2 vessel	13 (28.9%)	40 (19%)	
3 vessel	9 (20%)	45 (21.4%)	
LVEF (%)	54 ± 10	56 ± 12	0.22
Treatment at admission			
Aspirin	45 (100%)	207 (98.6%)	0.9
Clopidogrel	39 (86.7%)	161 (76.7%)	0.16
Nitrates	44 (97.8%)	202 (96.2%)	0.9
Statins	45 (100%)	207 (98.6%)	0.9
Angiotensin-converting enzyme inhibitors	18 (40%)	68 (32.4%)	0.9
*β*-Blockers	42 (93.3%)	191 (91%)	0.77
Biochemistry			
Creatinine (mg/dL)	1.17 ± 0.25	1 ± 0.87	0.25
Total cholesterol (mmol/L)	4.05 ± 1.04	4.00 ± 1.18	0.48
Peak troponin I (ng/mL)	4.85 ± 0.16	4.39 ± 0.17	0.09

Data is expressed as mean ± standard deviation and number of patients (%) for categorical variables.

MACE: major adverse cardiovascular events; LVEF: left ventricular ejection fraction.

**Table 2:** Inflammatory markers of non-ST-segment elevation acute coronary syndrome patients with and without MACE at 36-month followup.

Variable	MACE		*P* value
	Yes (*n* = 45)	No (*n* = 210)	
hsCRP at admission (mg/L)	7.4 [2.4–10.5]	7.4 [2.8–18.2]	0.79
hsCRP at 36-month followup (mg/L)	23 [10.4–34.7]	18.3 [7.3–26.6]	0.07
Delta hsCRP (mg/L)	15.5 [−28.7–73.3]	7.5 [−57.9–65.0]	0.01
GDF-15 at admission (pg/mL)	1639 [833–3151]	2190 [1333–3484]	0.09
GDF-15 at 36 months (pg/mL)	9105 [8071–9766]	3203 [2064–4572]	<0.001
Delta GDF-15 (pg/mL)	7605 [4831–8155]	602 [−405–2278]	<0.001

Data are expressed as median [interquartile range].

MACE: major adverse cardiovascular events; hsCRP: high-sensitivity C-reactive protein and GDF-15: growth-differentiation factor-15.

## References

[1] Sabatine MS, Morrow DA, de Lemos JA (2002). Multimarker approach to risk stratification in non-ST elevation acute coronary syndromes: simultaneous assessment of troponin I, C-reactive protein, and B-type natriuretic peptide. *Circulation*.

[2] Lagrand WK, Visser CA, Hermens WT (1999). C-reactive protein as a cardiovascular risk factor more than an epiphenomenon?. *Circulation*.

[3] Ridker PM (2003). High-sensitivity C-reactive protein and cardiovascular risk: rationale for screening and primary prevention. *The American Journal of Cardiology*.

[4] Shi Y, Massagué J (2003). Mechanisms of TGF-*β* signaling from cell membrane to the nucleus. *Cell*.

[5] Wollert KC, Kempf T, Lagerqvist B (2007). Growth differentiation factor 15 for risk stratification and selection of an invasive treatment strategy in non-ST-elevation acute coronary syndrome. *Circulation*.

[6] Kempf T, Sinning J-M, Quint A (2009). Growth-differentiation factor-15 for risk stratification in patients with stable and unstable coronary heart disease: results from the atherogene study. *Circulation: Cardiovascular Genetics*.

[7] Hochholzer W, Morrow DA, Giugliano RP (2010). Novel biomarkers in cardiovascular disease: update 2010. *American Heart Journal*.

[8] Domínguez-Rodríguez A, Abreu-González P (2009). Diurnal variations in biomarkers used in cardiovascular medicine: clinical significance. *Revista Española de Cardiología*.

[9] Dominguez-Rodriguez A, Tome MC, Abreu-Gonzalez P (2011). Inter-relation between arterial inflammation in acute coronary syndrome and circadian variation. *World Journal of Cardiology*.

[10] Fernández-Ortiz A, Pan M, Alfonso F (2012). Comments on the ESC guidelines for the management of Acute Coronary Syndromes in patients presenting without persistent ST-segment elevation. A report of the Task Force of the Clinical Practice Guidelines Committee of the Spanish Society of Cardiology. *Revista Española de Cardiología*.

[11] Dominguez-Rodriguez A, Abreu-Gonzalez P, Kaski JC (2009). Inflammatory systemic biomarkers in setting acute coronary syndromes—effects of the diurnal variation. *Current Drug Targets*.

[12] Wollert KC, Kempf T, Peter T (2007). Prognostic value of growth-differentiation factor-15 in patients with non-ST-elevation acute coronary syndrome. *Circulation*.

[13] Damman P, Kempf T, Windhausen F (2014). Growth-differentiation factor 15 for long-term prognostication in patients with non-ST-elevation acute coronary syndrome: an Invasive versus Conservative Treatment in Unstable coronary Syndromes (ICTUS) substudy. *International Journal of Cardiology*.

[14] Anand IS, Kempf T, Rector TS (2010). Serial measurement of growth-differentiation factor-15 in heart failure: relation to disease severity and prognosis in the valsartan heart failure trial. *Circulation*.

[15] Eggers KM, Kempf T, Wallentin L, Wollert KC, Lind L (2013). Change in growth differentiation factor 15 concentrations over time independently predicts mortality in community-dwelling elderly individuals. *Clinical Chemistry*.

[16] Kempf T, Zarbock A, Widera C (2011). GDF-15 is an inhibitor of leukocyte integrin activation required for survival after myocardial infarction in mice. *Nature Medicine*.

[17] Karakas M, Baumert J, Greven S, Rückerl R, Peters A, Koenig W (2010). Reproducibility in serial C-reactive protein and interleukin-6 measurements in post-myocardial infarction patients: results from the AIRGENE study. *Clinical Chemistry*.

